# 血浆宏基因组二代测序鉴定重型再生障碍性贫血血流感染病原体研究

**DOI:** 10.3760/cma.j.issn.0253-2727.2023.03.010

**Published:** 2023-03

**Authors:** 园 李, 佑祯 熊, 慧慧 樊, 丽萍 井, 建平 李, 青松 林, 春晖 徐, 英 李, 蕾 叶, 蒙 焦, 洋 杨, 洋 李, 文睿 杨, 广新 彭, 康 周, 馨 赵, 莉 张, 凤奎 张

**Affiliations:** 1 中国医学科学院血液病医院（中国医学科学院血液学研究所），实验血液学国家重点实验室，国家血液系统疾病临床医学研究中心，细胞生态海河实验室，天津 300020 State Key Laboratory of Experimental Hematology, National Clinical Research Center for Blood Diseases, Haihe Laboratory of Cell Ecosystem, Institute of Hematology & Blood Diseases Hospital, Chinese Academy of Medical Sciences & Peking Union Medical College, Tianjin 300020, China; 2 天津协和博精医学诊断技术有限公司，天津 301617 Microbiology Laboratory Tianjin Union Precision Medical Diagnostic Co., Ltd, Tianjin 301617, China

**Keywords:** 贫血，再生障碍性, 宏基因组, 二代测序, 血液, 感染, Anemia, aplastic, Metagenomic, Next-generation sequencing, Bloodstream, Infection

## Abstract

**目的:**

评估无细胞血浆宏基因组二代测序（mNGS）病原体识别对重型再生障碍性贫血（AA）血流感染的诊断意义。

**方法:**

应用mNGS与常规检测方法（血培养等）同步检测2021年2月至2022年2月中国医学科学院血液病医院贫血诊疗中心连续收治的29例AA患者共33例次送检样本，评估mNGS与常规检测的诊断一致性、对临床治疗获益的影响及临床准确度。

**结果:**

①33例次患者经mNGS和常规检测方法检测，其中25例次（75.76％）检出潜在病原微生物；共检出病原微生物72株，其中65株（90.28％）仅经mNGS检出。②诊断一致性评估：2例次（6.06％）组合符合（Composite），18例次（54.55％）mNGS唯一符合（mNGS only），2例次（6.06％）常规检测方法唯一符合（Conventional testing only），1例次（3.03％）共同符合（mNGS / Conventional testing），10例次（30.3％）完全不符合（None）。③临床治疗获益评估：8例次（24.24％）为启动靶向治疗（Initation of targeted treatment），1例次（3.03％）为降级治疗（Treatment de-escalation），13例次（39.39％）为确认治疗（Confirmation），11例次（33.33％）为无治疗获益（No clinical benefit）。④临床准确度：mNGS与常规检测方法差异有统计学意义（21/12对5/28，*P*<0.001），mNGS的敏感性80.77％，特异性70.00％，阳性预测值63.64％，阴性预测值84.85％，阳性似然比2.692，阴性似然比0.275。

**结论:**

mNGS不仅有助于精准诊断AA患者的血流感染，而且有利于指导进行精准抗感染治疗，临床准确度高。

## Abstract (en)

**Objective:**

To analyze the diagnostic value of cell-free plasma metagenomic next-generation sequencing（mNGS）pathogen identification for severe aplastic anemia（SAA）bloodstream infection.

**Methods:**

From February 2021 to February 2022, mNGS and conventional detection methods（blood culture, etc.）were used to detect 33 samples from 29 consecutive AA patients admitted to the Anemia Diagnosis and Treatment Center of the Hematology Hospital of the Chinese Academy of Medical Sciences to assess the diagnostic consistency of mNGS and conventional detection, as well as the impact on clinical treatment benefits and clinical accuracy.

**Results:**

①Among the 33 samples evaluated by mNGS and conventional detection methods, 25 cases（75.76％）carried potential pathogenic microorganisms. A total of 72 pathogenic microorganisms were identified from all cases, of which 65（90.28％）were detected only by mNGS. ②All 33 cases were evaluated for diagnostic consistency, of which 2 cases（6.06％）were Composite, 18 cases（54.55％）were mNGS only, 2 cases（6.06％）were Conventional method only, 1 case（3.03％）was both common compliances（mNGS/Conventional testing）, and 10 cases（30.3％）were completely non-conforming（None）. ③All 33 cases were evaluated for clinical treatment benefit. Among them, 8 cases（24.24％）received Initiation of targeted treatment, 1 case（3.03％）received Treatment de-escalation, 13 cases（39.39％）received Confirmation, and the remaining 11 cases（33.33％）received No clinical benefit. ④ The sensitivity of 80.77％, specificity of 70.00％, positive predictive value of 63.64％, negative predictive value of 84.85％, positive likelihood ratio of 2.692, and negative likelihood ratio of 0.275 distinguished mNGS from conventional detection methods（21/12 *vs* 5/28, *P*<0.001）.

**Conclusion:**

mNGS can not only contribute to accurately diagnosing bloodstream infection in patients with aplastic anemia, but can also help to guide accurate anti-infection treatment, and the clinical accuracy is high.

重型/极重型再生障碍性贫血（SAA/VSAA）患者由于严重而持久的粒细胞缺乏（粒缺），极易罹患致病微生物侵袭，炎症难以局限，常形成血流感染而缺乏明确感染灶，是患者最重要的致死原因之一[1]，及时有效地控制感染对于挽救生命以及获得良好预后均极为重要[2]。

病原学诊断始终是有效治疗感染最重要的环节。传统病原学检测模式是基于患者临床表现推测可能的感染类型，并相应地采用感染部位标本涂片染色、病理活检形态识别、免疫学检测和病原体培养等检测排查，这类检测方法耗时、费力、敏感性低，通常每种方法只对应少数病原体，覆盖面窄，易受治疗因素影响。SAA/VSAA常仅表现粒缺伴发热，无明确感染灶，或即使感染病灶明确，因血小板计数过低标本取材仍可能受限。

宏基因高通量二代测序（mNGS）进行病原学检测具备覆盖面宽、快捷精准、无偏倚、无创等优势，克服了常规病原学检测方法的局限，自2014年首次用于临床成功检出脑脊液钩端螺旋体感染[3]以来，在诊断血流感染、中枢神经系统感染、局灶性感染、呼吸道感染等多有应用[4]–[9]。目前，尚未见使用无细胞血浆检测SAA/VSAA血流感染的研究。我们对SAA/VSAA粒缺感染患者采用血浆mNGS识别致病微生物进行研究，以评估其临床实用价值。

## 病例与方法

一、病例资料

本研究为回顾性队列研究。以2021年2月至2022年2月中国医学科学院血液病医院贫血诊疗中心连续收治的29例AA患者共33例次感染/送检的样本为研究对象，其中31例次样本来自于27例粒缺伴感染的SAA/VSAA患者、2例次样本分别来自于1例输血依赖型非重型AA（TD-NSAA）患者和1例AA-阵发性睡眠性血红蛋白尿（PNH）综合征患者。AA诊断依据文献[10]标准。所有标本均在粒缺发热期进行取材，患者除贫血、严重血小板减少、粒缺外，尚表现发热（体温>38 °C）、畏寒等感染症状，并排除了肿瘤、结缔组织病、代谢等因素相关发热。对所有患者在发热/畏寒时抽取静脉血样进行常规病原学检测（血培养等）及mNGS检查。

二、血培养检测

患者的血液标本使用FX400全自动血培养仪（BD，美国）、VITEK2 compact细菌鉴定仪（生物梅里埃，法国）进行微生物学检测及药敏实验（MIC法）分析。病原微生物分离培养与鉴定严格符合《全国临床检验操作规程》。

三、mNGS检测

mNGS对可疑的病原微生物进行DNA宏基因组检测，可检测范围包括基因组序列已知的12 895种细菌、11 120种病毒、1 582种真菌、312种寄生虫、177种分枝杆菌复合群（11个结核分枝杆菌复合群、166个非结核分枝杆菌）和184种支原体/衣原体。使用1901#核酸提取/纯化试剂提取DNA；使用2012A#病原微生物核酸检测试剂盒（MGI-DNA）进行片段化、DNA末端修复、接头连接和PCR扩增及产物纯化；构建好的文库经过Pooling之后，使用MGI-2000/200测序仪进行测序，测序长度SE50，测序数据量平均不低于30 M Reads。通过Genseq-PM软件进行病原微生物测序数据分析，用BWA与微生物基因组数据库（从NCBI中筛选组成）进行比对，从而对微生物进行鉴定。参照文献[11]的界值：当细菌序列数（reads）≥10、病毒序列数（特定基因组区域）≥3、或真菌序列数≥2时，认为mNGS检测该种病原微生物为阳性。

四、临床分析

1. 临床分析方法：由本中心至少3位资深临床医师、微生物学专家和生物信息分析专家基于患者的临床特点、病原微生物的致病特点、影像学特点、常规病原学检测结果、mNGS检测结果、抗感染疗效等进行综合审议，确定最终临床诊断（FCD）和责任病原微生物。

2. 诊断一致性评估：以FCD为标准，将mNGS和常规检测方法的检测结果分别进行比对，评估一致性。一致性级别分为5级：①共同符合（mNGS / Conventional testing）：两种检测方法的结果都分别与FCD完全一致；②mNGS唯一符合（mNGS only）：仅mNGS检出的结果与FCD一致；③常规检测方法唯一符合（Conventional testing only）：仅常规检测方法检出的结果与FCD一致；④组合符合（Composite）：两种检测方法的结果都与FCD部分一致，须组合在一起才与FCD完全一致；⑤完全不符合（None）：mNGS和常规检测方法的结果均与FCD不一致。

3. mNGS对于临床治疗获益的评估：依据mNGS检出的病原学结果对于临床抗感染治疗的指导情况，作出临床治疗获益评估。分为4级：① 启动靶向治疗（Initation of targeted treatment）：仅mNGS检出了与当时感染相关的某种病原微生物，相应地采用了针对该种病原微生物的靶向治疗；② 降级治疗（Treatment de-escalation）：依据mNGS的病原微生物检测结果，相应地进行了降阶梯治疗或者结束治疗；③ 确认治疗（Confirmation）：依据mNGS的检测结果，确认了现行的抗感染治疗方案并继续进行；④ 无治疗获益（No clinical benefit）：依据常规检测方法的病原学结果或者仅依据医师临床经验，调整抗感染治疗。其中①、②和③三种级别属于实质的临床治疗获益。当患者存在针对于多种病原学结果的多种临床治疗获益级别时，优先选择顺序设定为：启动靶向治疗>降级治疗>确认治疗>无治疗获益。

五、统计学处理

采用SPSS 23.0软件进行统计学分析。连续变量不符合正态分布，以中位数（*IQR*）描述；分类变量以频数和百分比表示，组间比较采用Fisher精确概率法。通过受试者工作特征曲线（ROC曲线）计算诊断的敏感性、特异性、阳性预测值、阴性预测值、阳性似然比及阴性似然比。双侧*P*<0.05为差异有统计学意义。

## 结果

1. 患者基本特征：29例患者中，男13例，女16例，中位年龄51（23.5，57）岁。33例次送检样本时患者血常规：中位中性粒细胞绝对值（ANC）0.11（0.03，0.37）×10^9^/L，中位HGB 68（57，78）g/L；中位PLT 18（8，23）×10^9^/L，中位网织红细胞绝对值（ARC）0.005（0.002，0.013）×10^9^/L。血生化：中位血清白蛋白（ALB）浓度30.6（28.5，33.6）g/L，中位球蛋白（GLO）浓度28.5（24.0，32.0）g/L。临床怀疑和明确部位的感染共23例次，包括：颅内感染（1例次）、颜面软组织感染（1例次）、齿龈感染（1例次）、肺炎（15例次）、消化道感染（2例次）、肺炎并颌下淋巴结炎（1例次）、肺炎并齿龈感染（1例次）、齿龈感染并颜面软组织感染（1例次）；无可识别感染部位的粒缺发热10例次。

2. 病原微生物检出情况：33例次样本经mNGS和常规检测方法检测，共计检出潜在致病微生物72株，其中65株（90.28％）仅经mNGS检出（分别为细菌22株，真菌15株，病毒28株），2株（2.78％）仅经常规检测方法检出（均为细菌），5株（6.94％）经mNGS和常规检测方法两种方法均有检出（均为细菌）。72株潜在致病微生物，包括细菌29株，真菌15株，病毒28株。细菌以嗜麦芽窄食单胞菌、大肠埃希菌、肺炎克雷伯菌为主，真菌以烟曲霉菌（4株）、黄曲霉菌（2株）、米根霉菌（2株）和微小根毛霉菌（2株）为主，病毒以EB病毒（EBV）、巨细胞病毒（CMV）、人类单纯疱疹病毒1型（HSV1）为主。临床少见或特殊病原微生物如嗜肺军团菌（1株）、蜡样芽胞杆菌（2株）、米根霉菌（1株）、微小根毛霉菌（1株）、耶氏肺孢子虫菌（1株）等经mNGS检出。解鸟氨酸拉乌尔菌（1株）和阴沟肠杆菌（1株）未经mNGS检出，经由常规检查方法检出。全部33例次感染/送检样本中，25例次（75.76％）检出潜在致病微生物，4例次（12.12％）检出1株，10例次（30.30％）检出2株，7例次（21.21％）检出3株，4例次（12.12％）检出4株及以上。

3. 诊断一致性评估：对全部33例次样本进行了诊断一致性评估，其中2例次（6.06％）为Composite，18例次（54.55％）为mNGS only，2例次（6.06％）为Conventional testing only，1例次（3.03％）为mNGS / Conventional testing，10例次（30.3％）为None。提示常规检测方法会漏诊54.55％的感染，这部分经mNGS明确诊断。

对全部72株潜在致病微生物进行了一致性评估，其中42株（58.33％）为责任致病微生物，30株（41.67％）为非责任致病微生物。42株责任致病微生物包括：mNGS only 35株（83.33％），mNGS / Conventional testing 5株（11.90％），Conventional testing only 2株（4.76％）。35株mNGS only致病微生物分别为20株细菌和15株真菌，5株mNGS / Conventional testing和2株Conventional testing only致病微生物均为细菌。30株非责任致病微生物分别是2株细菌和28株病毒。通过mNGS诊断，细菌性致病微生物与临床最终诊断的一致性为86.21％（25/29），真菌性致病微生物的一致性为100.00％（15/15），病毒性致病微生物的一致性为0（0/28）。

4. mNGS对于临床治疗获益的评估：对全部33例次样本进行了临床治疗获益评估。其中，8例次（24.24％）为启动靶向治疗，1例次（3.03％）为降级治疗，13例次（39.39％）为确认治疗，11例次（33.33％）为无治疗获益。8例次启动靶向治疗的感染，涉及的病原微生物共12株，分别为蜡样芽胞杆菌（1株）、嗜麦芽窄食单胞菌（4株）、阴沟肠杆菌（1株）、嗜肺军团菌（1株）、耶氏肺孢子虫菌（1株）、烟曲霉菌（1株）、小孢根霉菌（1株）、微小根霉菌（1株）及米根霉菌（1株）。

对经mNGS检出的全部70株潜在致病微生物进行了临床治疗获益的评估。27株细菌性致病微生物的临床治疗获益分别为：8株（29.63％）为启动靶向治疗，16株（59.26％）为确认病原菌鉴定/治疗，1株（3.70％）为降级治疗，2株（7.41％）为无临床获益；15株真菌性致病微生物的临床治疗获益分别为：5株（33.33％）为启动靶向治疗，10株（66.67％）为确认病原菌鉴定/治疗；28株病毒性致病微生物的临床治疗获益为：28株（100.00％）为无临床获益。

5. mNGS的临床准确度分析：对全部33例次样本的mNGS进行临床准确度分析。mNGS结果与最终临床诊断一致者为21例次，不一致者12例次，而常规检测方法分别为5例次和28例次。差异有统计学意义（*P*<0.001）。mNGS的敏感性80.77％，特异性70.00％，阳性预测值63.64％，阴性预测值84.85％，阳性似然比2.692，阴性似然比0.275。

## 讨论

本研究中，我们评估了无细胞血浆mNGS在AA患者血流感染中的临床诊断价值和治疗指导作用。无细胞血浆mNGS除具备无创、快速精准、全面等技术优势外，还能避免某些胞内菌或胞内病毒等微生物干扰检测结果，降低假阳性的出现，这些技术特点符合SAA/VSAA患者血流感染的诊断需求。经评估，我们发现mNGS在这方面较常规检测方法敏感性高，可更全面地检出潜在致病菌，诊断优势显著，且可与常规检测方法优势互补，mNGS对抗感染治疗有重要的指导作用，增加临床治疗获益。

本研究的方法学中，mNGS借鉴的阳性判读标准来源于针对于脑脊液样本的相关研究[11]，基于4个方面考虑：①阳性判读标准应明确、简易、可行性强；②符合本研究要求的且适合无细胞血浆样本的阳性判读标准罕见报道；③通常认为宿主血浆是无菌的，类似于脑脊液、关节液等，不存在定植菌；④有报道[12]发现血浆阶段性出现细菌DNA，提示可能存在血液定植菌，即DNA血症（DNAemia），不同于健康志愿者以双歧杆菌等厌氧菌为主，发热患者以需氧菌或微嗜氧菌为主，那么，在对无细胞血浆样本的阳性判读时需尽力排除定植菌因素干扰。因此，本研究借鉴了脑脊液样本研究的阳性判读标准。相信随着研究的增多和深入，适合无细胞血浆样本研究的阳性判读标准会逐渐明晰。

本研究中，mNGS于25例次（75.76％）样本中检出潜在致病微生物，高于常规检测方法（5例次，15.15％），且其中21例次（63.64％）为多种病原微生物混合感染，常规检测方法仅检出1例混合感染（阴沟肠杆菌混合解鸟氨酸拉乌尔菌），这与Guo等[13]的研究发现一致（分别为85.7％、38.8％、51.0％、0），也符合Blauwkamp等[14]和Chen等[9]的观点。与Valdez等[2]研究相比，本研究中病原微生物的分布趋势也出现了变化，细菌方面，嗜麦芽窄食单胞菌和肺炎克雷伯菌趋于占主导优势，几乎未检测出革兰阳性菌，这与《中国中性粒细胞缺乏伴发热患者抗菌药物临床应用指南（2020年版）》[15]一致，指南认为粒缺伴发热患者的责任致病微生物主要是革兰阴性菌。但Li等[16]和Martin等[17]的观点有差异，研究间的背景时期、目标人群构成、基础疾病构成、疾病状态的不同可能造成了该差异，Martin等[17]的研究反映了1979−2000年美国全国发热患者的病原学特征，基础疾病以糖尿病、癌症、慢性心衰、慢性阻塞性肺病为主，粒缺或免疫缺陷的患者比例较低，这与我国SAA/VSAA患者的疾病背景状态迥然不同；曲霉菌目（7株）排在侵袭性真菌感染的首位，接合菌纲（毛霉科和根霉科）仍排在第二位，但发生（5株）趋于增多，几乎接近于曲霉菌目，远高于Valdez等[2]的报道（接合菌纲3/30）。本研究中，这种病原微生物分布的改变可能与重视细菌和真菌（曲霉菌目）感染的预防性治疗，以及广谱抗生素对病原微生物的筛选作用有关。此外，临床少见或特殊病原微生物如嗜肺军团菌、耶氏肺孢子菌等也经mNGS检出，在以往的AA患者中报道较少。病毒类微生物多有检出，但经临床综合审议，确定为非致病微生物，未进入最终临床诊断，属于假阳性。因此，在检测AA患者的潜在病原微生物方面，mNGS较常规检测方法更敏感，其优势主要体现在细菌和真菌方面。说明mNGS更有助于诊断SAA/VSAA患者的细菌性或真菌性感染并发症。mNGS的这种优势取决于该方法学的工作机制。基于目标基因的集合、文库制备时目标基因的桥接扩增和片段化形成及剪接等原理，mNGS可高通量检测血浆中的游离的目标基因DNA片段或RNA片段，从而同时精准鉴定多种致病微生物，且不受抗生素应用的影响。相较于病毒，细菌和真菌分子量及体积较大，应用PCR等一代测序方法鉴定存在耗时长、菌谱覆盖窄等问题，因而，传统检测方法鉴定细菌或真菌通常是通过鉴定完整的菌落以得出结论，但培养菌落的阳性率低，且易受到抗生素使用的影响，不能适应临床需求。mNGS在生信分析时需要比较去除人源核酸片段，并排除定植菌，区分病原菌和污染菌，这需要mNGS根据实验室自身特点、标本特点、检测条件批次间特点做个体化处理。

本研究中，mNGS使得更多患者明确了感染的病原学诊断，在常规检测方法的基础上，增加了20例次（60.61％），包括2例次Composite和18例次mNGS only，提高了诊断效率。这与Grumaz等[4]和Guo等[13]的研究结果相似（分别为53％和46.9％），高于Hogan等[5]的研究（7.3％）。说明mNGS有助于更全面完整地明确诊断。当然，分别有2例次Composite和2例次常规检测方法Conventional testing only，说明mNGS并不能解决所有情况下的感染诊断，需要与常规检测方法互补使用。本研究中，嗜肺军团菌、耶氏肺孢子菌等少见类型感染的诊断为mNGS only，混合感染的诊断主要为mNGS only，本研究说明在诊断少见类型感染和混合感染方面，mNGS可令患者获益。Chen等[9]的研究也认同mNGS可使临床获益；Peng等[18]尽管确认55％的患者为混合感染，但是认为mNGS与常规微生物检测方法的临床诊断价值差异无统计学意义（61.7％对76.7％，*P*＝0.11），这也许是与标本类型以及采集标本方法有关。

本研究中，10例次（30.3％）None检出的潜在致病微生物共30株，造成这些不符合的可能原因有进行mNGS检测前血浆的无细胞纯化程度、存在定植菌或污染菌、mNGS生信分析的技术局限等。

精准的诊断带动了治疗获益。Zhan等[8]研究显示，启动靶向治疗者高达28％。本研究中，22例次样本因mNGS受益，其中8例次（24.24％）及时安排了靶向抗感染治疗。与上述研究相近。在嗜肺军团菌等特殊类型感染方面，mNGS不仅确立了精准治疗方向，而且有助于制订个体化治疗方案。mNGS在13例次（39.39％）确认治疗者的作用主要体现在确认速度快。

本研究中，mNGS的临床准确度分析表明mNGS较常规检测方法更敏感，适于筛查，有助于临床检出更多潜在的致病微生物，降低漏诊风险，同时，mNGS的特异性偏低，阴性预测值偏高，阴性结果可利于排除某些潜在的病原微生物。mNGS的阳性似然比和阴性似然比结果，以及2例次（6.06％）的常规检测方法Conventional testing only，提示mNGS不足以完全替代常规检测方法，两种方法都需临床应用，优势互补。虽然Hogan等[5]和Parize等[19]的研究与本研究的设计有所不同，但也都认为mNGS检测血液样本较常规检测方法敏感性高（分别为71.43％、36％），阴性预测值高（分别为85.71％、98.4％），与本研究的结论相似。关于mNGS假阴性（常规检测方法检测阳性）的情况，推测可能与血样中相关病原微生物载量偏低且宿主细胞过多有关。因而，两种方法都需临床应用、优势互补。此外，在mNGS用于诊断血流感染方面还存在一些亟待解决的问题，例如，mNGS诊断血流感染的最适应用时间窗、mNGS结果的解读规范等。

本研究存在一些不足：①样本量较少；②治疗中应用广谱抗生素较普遍，可能影响常规检测方法的效能以及细菌感染诊断的判定；③患者基础感染病灶部位的确切病原学证据较少，可能会影响病原微生物的致病责任判定。在后续的工作中，我们将克服上述不足，设计高等级的临床研究，以解决上述问题。

总之，本研究展示了mNGS的临床价值，不仅可精准诊断AA患者的血流感染，而且能指导更多患者接受精准抗感染治疗，可常规用于诊治AA患者的血流感染。
